# Integrative “Omic” Analysis for Tamoxifen Sensitivity through Cell Based Models

**DOI:** 10.1371/journal.pone.0093420

**Published:** 2014-04-03

**Authors:** Liming Weng, Dana Ziliak, Bonnie LaCroix, Paul Geeleher, R. Stephanie Huang

**Affiliations:** Section of Hematology and Oncology, Department of Medicine, University of Chicago, Chicago, Illinois, United States of America; Dartmouth, United States of America

## Abstract

It has long been observed that tamoxifen sensitivity varies among breast cancer patients. Further, ethnic differences of tamoxifen therapy between Caucasian and African American have also been reported. Since most studies have been focused on Caucasian people, we sought to comprehensively evaluate genetic variants related to tamoxifen therapy in African-derived samples. An integrative “omic” approach developed by our group was used to investigate relationships among endoxifen (an active metabolite of tamoxifen) sensitivity, SNP genotype, mRNA and microRNA expressions in 58 HapMap YRI lymphoblastoid cell lines. We identified 50 SNPs that associate with cellular sensitivity to endoxifen through their effects on 34 genes and 30 microRNA expression. Some of these findings are shared in both Caucasian and African samples, while others are unique in the African samples. Among gene/microRNA that were identified in both ethnic groups, the expression of *TRAF1* is also correlated with tamoxifen sensitivity in a collection of 44 breast cancer cell lines. Further, knock-down *TRAF1* and over-expression of hsa-let-7i confirmed the roles of hsa-let-7i and *TRAF1* in increasing tamoxifen sensitivity in the ZR-75-1 breast cancer cell line. Our integrative omic analysis facilitated the discovery of pharmacogenomic biomarkers that potentially affect tamoxifen sensitivity.

## Introduction

Tamoxifen (TAM) is a selective estrogen receptor (ER) modulator that has been used to treat and prevent breast cancer for 30 years [1]. In clinics, one of the biggest concerns regarding TAM therapy is that approximately 35% patients do not respond to TAM treatment [2]. Moreover, patients taking TAM medication have exhibited a large variety of side effects, such as hot flashes and osteoporosis, which varied greatly from patient to patient [3]. All these highlight the need of conducting pharmacogenomic research to identify biomarkers so that patient’s responses to TAM therapy could be predicted and treatment outcomes could be improved.

So far, most of the pharmacogenomic research on TAM therapy has been conducted in patients with Caucasian ancestry. However, ethnic differences were observed between Caucasian- and African-derived populations both in breast cancer incidence and treatment outcomes. One report showed that African-American patients were at lower risk to develop breast cancer but displayed higher rate of mortality, compared to Caucasian Americans [4]. Further, African American patients appeared to have lower incidence in ER-positive breast cancer than Caucasian Americans [5].These ethnic differences highlight the need to conduct pharmacogenomics research in African-derived population.

Since ER regulates hundreds of downstream gene expressions [6], as well as cross-talks with other signaling pathways, such as G-protein coupled receptor and Ras/MAPK signaling pathways [7], inactivation of ER would cause interruptions of many physiologically important processes. We hypothesize that an integrative approach that evaluated multiple genetic/expression components all at once is more likely to yield biologically relevant biomarkers for TAM sensitivity. Recruiting patients in clinical trials has become increasingly expensive. Therefore, we sought to use a cell-based model to achieve this goal. Indeed, the International HapMap lymphoblastoid cell lines (LCLs) that provides publicly available genome-wide genetic and other omics data, have been utilized to successfully identify multiple novel pharmacogenomic markers [8]. Excitingly, a number of these HapMap samples derived markers have been validated in clinical studies as treatment outcome predictors [9]–[11]. More importantly, our previous work has shown that ER-alpha was expressed and functional in randomly selected LCLs [10]. In this study, we set out to comprehensively evaluate genetic/transcriptomic/epigenomic (in the form of microRNA (miRNA)) contribution of TAM sensitivity in a set of HapMap YRI LCLs. Since the majority of CYP450 enzymes are poorly expressed in LCLs, including CYP2D6, a major enzyme that converts TAM into its active metabolite endoxifen, we used endoxifen for phenotyping in our initial LCL-based discovery model.

## Materials and Methods

### Cell Lines and Materials

The International HapMap LCLs were purchased from the Coriell Institute for Medical Research (Camden, NJ). Specifically, LCLs derived from 58 unrelated individuals from Yoruba in Ibadan, Nigeria (YRI, HAPMAPPT03) were used for genome-wide discovery study. The HapMap LCLs were derived from multiple ethnic groups. In this study, we chose to focus on African derived samples (58 YRI LCLs) due to the existing observation on ethnic differences as well as the need of conducting research in underrepresented population. LCLs were maintained in RPMI 1640 supplemented with 1% L-glutamine from Mediatech (Herndon, VA) and 15% fetal bovine serum (FBS) from Hyclone (Logan, UT). Cells were diluted three times per week and seeded at a concentration of 350,000 cells/ml at 37°C in a 95% humidified 5% CO_2_ atmosphere.

The breast cancer cell line, ZR-75-1, was purchased from ATCC (www.atcc.org) and maintained in RPMI 1640 supplemented with 10% FBS. This breast cancer cell line was passaged to 20∼30% confluency (∼6×10^3^ cells/cm^2^) three times a week and maintained at 37°C in 95% humidified atmosphere with 5% CO_2_.

TAM was purchased from Sigma-Aldrich. Endoxifen, an active metabolite of TAM, was a generous gift from Dr. David Flockhart (Indiana University). Phenol red free RPMI was purchased from Mediatech and charcoal stripped FBS was purchased from Gemini Bio products (Sacramento, CA). Ethanol used to make endoxifen stocks was purchased from Decon laboratories, Inc. (King of Prussia, PA).

### Phenotyping Cellular Sensitivity to Endoxifen in YRI LCLs

We have previously developed a method for phenotyping cellular sensitivity to endoxifen in HapMap LCLs [10]. This method was applied to evaluate cellular sensitivity to endoxifen in 58 unrelated HapMap YRI LCLs. Specifically, each HapMap LCL sample was transferred into “hormone-free” media (phenol red free RPMI1640, 15% charcoal stripped FBS, and 1% L-glutamine) 72 hours prior to the experiment. The LCLs that displayed >85% viability were pelleted and resuspended in the new “hormone-free” media at a concentration of 350,000 cells/ml. These LCLs were plated at a concentration of 10,000 cells/well in hormone-free media in triplicate in a 96-well round bottom plate (Becton Dickinson Labware, Franklin Lakes, NJ). Twenty-four hours later, increasing concentrations of endoxifen (3, 5, 7, 10 μM) were added to these LCLs, incubating for another 72 hours. AlamarBlue (Biosource, Camarillo, CA) was added 24 hours before growth inhibition evaluation using the Synergy-HT multi-detection plate reader (BioTek, Winooski, VT). The percent survival at each treatment concentration compared to control was obtained for each cell line via the manufacturer’s protocol [12].

### Identification of Genetic Predictors using a Genome-wide Integrative “omic” Strategy

Our lab has developed a genome-wide integrative approach which integrates drug sensitivity, SNP genotype, transcriptional gene expression and miRNA expression to identify pharmacogenomic predictors of drug sensitivity (a separate manuscript under review). We applied this novel method to identify genetic predictors for endoxifen sensitivity in HapMap YRI samples, resulting in 6 steps of associations among 4 datasets, as detailed in Figure S1. SNP genotypes were downloaded from the International HapMap database (www.HapMap.org) (release 28). Genome-wide gene expression was assessed by Affymetrix GeneChip Human Exon 1.0 ST array [13]. Data were obtained through GEO (GSE# 7761). miRNA expression was assessed with Exiqon miRCURY LNA arrays v.10.0 and data obtained from GEO (GSE# 34406). For each of the endoxifen sensitivity phenotype [log-transformed percent viable cells at each endoxifen treatment concentration], a sequential multi-steps association analysis was conducted. The purpose of phenotypic data log transformation is to satisfy the normal distribution assumption of subsequent linear regression analysis. In step 1, genome-wide correlation test was performed between more than 13,000 gene/transcript cluster expression and 4 endoxifen phenotypes [log-transformed percent viable cell after 3, 5, 7, 10 μM endoxifen treatment] independently. All genes whose expression levels were correlated with at least one endoxifen sensitivity phenotype at p<0.05 were brought to subsequent analysis. Step 2, general linear regression was run between levels of 201 LCL-expressed miRNAs [14] and 4 endoxifen phenotypes, with p<0.05 as the filtering criteria. Step 3, negative expression correlations between genes and miRNA identified in step 1 and 2 were examined by using the SCAN database (www.scandb.org/apps/microRNA), a public available online resource built by our group to host relationships data among genetic/mRNA/miRNA expression [14], [15]. The threshold used was p≤10^−4^ (equivalent to false discovery rate less than 0.05). In step 4, association between SNP genotype and gene expression passed step 3 filtering was examined by using SCAN database (http://www.scandb.org/newinterface/index.html) using cutoff of p≤10^−4^. Step 5, the associated SNPs identified from step 4 were further investigated for their association with miRNA expression identified from step 3 (p<0.05). Step 6, the SNPs associated with both gene (step 4) and miRNA (step 5) expressions were submitted for a GWAS analysis against each endoxifen sensitivity phenotype independently (p≤10^−4^). In Step 4, 5 and 6, additive genetic model was assumed. All linear regression tests were performed using (lm) function in an R package.

### Evaluation of the Candidate Gene Expression in 44 Breast Cancer Cell Lines

The expression of 34 candidate genes generated by our integrative omic model was analyzed for their correlation with log_10_ TAM (GI_50_) data from the 44 breast cancer cell lines, with p<0.05 as the cutoff. Both gene expression and log_10_ TAM (GI_50_) data were retrieved from a previous publication [16]. Briefly, gene expression was assessed using the Affymetrix GeneChip Human Gene 1.0 ST exon array platform. Dose-response curve used to estimate GI_50_ was generated by treating cancer cells with 9 doses of TAM with 1∶5 serial dilutions in triplicates for 72 hours. Cell proliferation was measured using Cell Titer-Glo assay (Promegma, WI). Dose-response data were then fit into Gompertz curve with nonlinear least squares procedure.

### Functional Evaluation of hsa-let-7i and *TRAF1* in a Breast Cancer Cell Line

#### Gene/miRNA modification

siRNA knock-down of *TRAF1* gene and over-expression hsa-let-7i using a mimic were performed in ZR-75-1 breast cancer cell line using DharmaFECT transfection kit (Thermo Scientific). Specifically, 1000 cells/well ZR-75-1 were plated in 96-well plates 24 hours prior to experiment. DharmaFECT transfection reagent 1 was used to transfer siTRAF1 and hsa-let-7i mimic into the cells. siRNAs for *TRAF1* (cat. # SI03077606), let-7i-5p mimic (cat.# MSY0000415) and scramble control siRNA (cat. # 1027292) were purchased from Qiagen (Valencia, CA). 100 μl transfection media that contain 25 nM siRNA or mimics, 0.2 μl DharmaFECT transfection reagents 1 and the corresponding growth media were added to each well of the 96-well plate. 24 hours later, transfection media were removed and replaced with regular growth media containing 0, 2, 4, 8, 12, and 16 μM TAM and incubated for 48 hours. Cell viability was measured using CellTiter-Glo, a luminescent cell viability assay (Promega). Luminescence at 100 nm was read using the Synergy-HT multi-detection plate reader (BioTek). Percentage of cell survival rates was calculated using raw luminescence values between TAM treated cells and those of control wells. In addition, the siRNA knock down or mimic overexpression was confirmed by RT-PCR after 24 hours of transfection.

#### Real time PCR quantification of *TRAF1* and hsa-let-7i

Quantitative real time PCR method was employed to evaluate the changes of gene or miRNA expression upon knocking down or overexpression in the breast cancer cell line. Qiagen RNeasy Plus Mini Kit (Valencia, CA) was used for lysing the cells and extracting RNAs. The amount of total RNA was measured on Nanodrop 8000 (Thermo Scientific, Waltham, MA). 1 μg of total RNA was used to construct cDNA library of mRNA by reverse transcription with the high-capacity cDNA Transcription Kit (Applied Biosystems, Foster city, CA), while 20 ng of total RNA was used for cDNA library of miRNA with miRCURY Universal cDNA synthesis kit (Exiqon, Woburn, MA). For qPCR reaction, 5 and 0.05 ng cDNA templates were used respectively for *TRAF1* and hsa-let-7i amplifications in 10 μl volume. The primers for amplifying *TRAF1* were purchased from Applied Biosystems (cat # Hs01090170-m1). The primers for amplifying hsa-let-7i-5p were purchased from Exiqon, (cat # 204394-01). cDNA mixtures for making standard curve was prepared by pooling cDNA from all samples tested with a series of dilutions of 1∶4. A house-keeping gene, B2M, was used as the internal control for quantification of mRNA expression. B2M forward primer: GATGAGTATGCCTGCCGTGTG; B2M reverse primer: CAATCCAAATGCGGCATCT (Applied Biosystems). U6 snRNA was used as housekeeping for quantification of miRNA (Exiqon, lot # 119008). The PCR reactions were performed in either TaqMan Fast Advanced Master Mix (Applied Biosystems, lot # 1106010), or in Power SYBR Green PCR Mastermix (Applied Biosystems, cat # 4368577), using ABI ViiA7 (Applied Biosystems). Relative gene expression was calculated as the ratio of the quantity of gene of interest to the quantity of the housekeeping gene using the standard curves.

## Results

### Cellular Sensitivity to Endoxifen in YRI LCLs

As stated in our previous publication [10], we used endoxifen instead of TAM for cellular phenotyping for the reason that LCLs do not express high levels of *CYP2D6* and other P450 enzymes. A modified AlarmaBlue assay was employed to measure endoxifen induced growth inhibition by using phenol red free RPMI culture media and charcoal stripped FBS as supplement. Growth inhibition caused by endoxifen treatment was measured at 4 concentrations (3, 5, 7 and 10 μM) in the 58 HapMap YRI samples.

**Figure 1:**
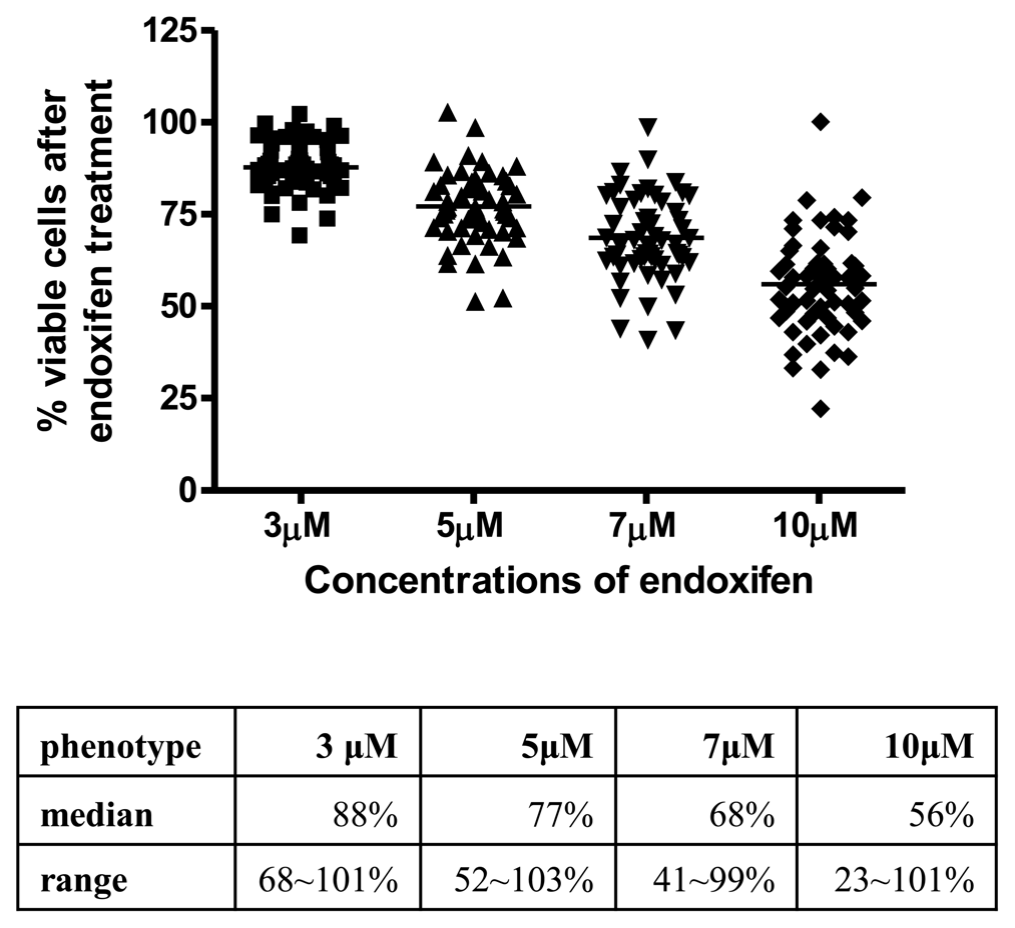
Scatter plot of endoxifen sensitivity in YRI LCLs. X axis represents the 4 treatment concentrations of endoxifen (3, 5, 7 and 10 μM) and Y axis represents percent viable cells 72 hours after drug treatment. The table underneath shows median value and data range at each treatment condition.

As expected, a dose-dependent cellular growth inhibition was observed with increasing concentrations of endoxifen treatment in YRI LCLs, with median percent viable cells decreased from 88% to 56% after 3 and 10 uM endoxifen 72 hours treatment. Moreover, the inter-individual variations appear to be larger at higher treatment concentrations, as shown in Figure 1.

### Genome-wide Identification of Markers for Endoxifen Sensitivity in YRI LCLs

An integrative omic approach developed by our lab was used to identify genetic variants to endoxifen sensitivity, which involves 6 steps of sequential association tests among 4 datasets: cellular sensitivity to endoxifen, whole genome SNP genotype, mRNA expression and miRNA expression (Figure S1). After these sequential integrative analyses, we identified a handful of SNPs, miRNAs and mRNAs that associate to endoxifen sensitivity but also are inter-correlated. The number of findings is shown in Table 1. Taken all phenotypes together, we identified a total of 141 unique associations involved with 50 SNPs, 34 genes and 30 miRNAs related to endoxifen sensitivity (Table S1).

**Table 1:** Summary findings of integrative omic analysis of endoxifen sensitivity in HapMap YRI LCLs.

Phenotype	3 μM	5 μM	7 μM	10 μM
Step1: pheno∼mRNA (p<0.05)	788 genes	897 genes	846 genes	1117 genes
Step2: pheno-miRNA (p<0.05)	32 miRNAs	71 miRNAs	74 miRNAs	94 miRNAs
Step3: mRNA∼miRNA (p≤0.0001)	13 miRNAs	33 miRNAs	45 miRNAs	56 miRNAs
	28 genes	87 genes	95 genes	119 genes
Step 4: mRNA∼SNP (P≤0.0001)	28 genes	87 genes	95 genes	119 genes
	18410 SNPs	45943 SNPs	47725 SNPs	45477 SNPs
Step 5: SNP∼miRNA (p<0.05)	13 miRNAs	33 miRNAs	45 miRNAs	56 miRNAs
	9080 SNPs	33044 SNPs	35362 SNPs	36418 SNPs
Step 6: SNP∼pheno (p≤0.0001)	4 SNPs	22 SNPs	29 SNPs	23 SNPs
	10 miRNAs	31 miRNAs	42 miRNAs	50 miRNAs
	5 genes	84 genes	92 genes	117 genes
Final Associations	2 SNPs	16 SNPs	26 SNPs	11 SNPs
	4 miRNAs	18 miRNAs	21 miRNAs	10 miRNAs
	3 genes	14 genes	16 genes	13 genes

Six steps of associations among endoxifen sensitivity, SNP genotype, mRNA and miRNA expressions were conducted sequentially for 4 endoxifen treatment concentrations independently**. Step 1**, the association between the phenotypes of endoxifen sensitivity and ∼13,000 gene/transcript cluster expressions; **step 2**, the association between the phenotypes of endoxifen sensitivity and 201 miRNA expressions; **step 3**, the negative correlation between mRNA and miRNA expressions from step 1 and step 2; **step 4**, the association between mRNA expressions from step 3 and SNP genotypes; **step 5**, the association between miRNA expression from step 3 and SNP genotypes from step 4; **step 6**, the association of SNP genotypes from step 5 and the phenotypes of endoxifen sensitivity. The final associations are the SNP genotypes, mRNA and miRNA expressions from the step 6 that correlate with endoxifen sensitivity but also associate each other. Numbers of identified biomarkers were indicated in each step for each phenotype. “3, 5, 7 and 10 μM” represent phenotypes of the 4 concentrations of endoxifen treatment. “Pheno” represents the phenotypes of endoxifen sensitivity.

**Figure 2:**
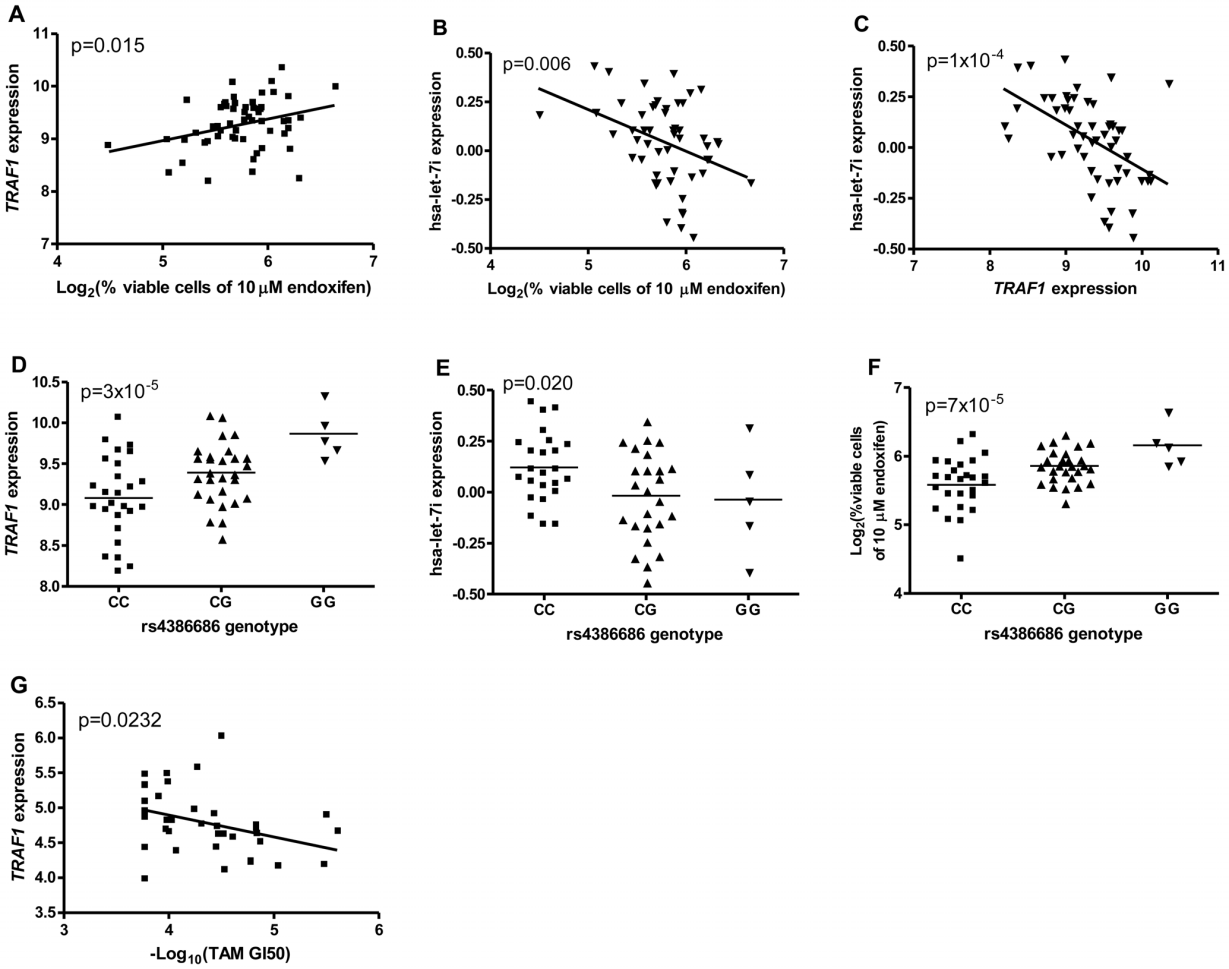
Relationships of endoxifen/TAM sensitivity, rs4386686, hsa-let-7i and *TRAF1* expression. **A)** Step1: association between *TRAF1* expression and percent viable cells after 10 μM endoxifen treatment in YRI LCLs; **B)** Step2: association between hsa-let-7i expression and percent viable cells after 10 μM endoxifen treatment in YRI LCLs; **C)** Step 3: negative correlation of *TRAF1* and hsa-let-7i expression in YRI LCLs; **D)** Step 4: association between *TRAF1* expression and rs4386686 genotypes in YRI LCLs; **E)** Step 5: association between hsa-let-7i expression and rs4386686 genotypes in YRI LCLs; **F)** Step 6: association between rs4386686 and percent viable cells after 10 μM endoxifen treatment in YRI LCLs; **G)** association between *TRAF1* expression and -log_10_(TAM GI_50_) in 44 breast cancer cell lines.

A representation of findings from this integrative omic analysis is exemplified by associations among rs4386686, hsa-let-7i and *TRAF1* to endoxifen sensitivity (Figure 2). *TRAF1* expression and phenotype (% cell growth inhibition after 10 μM endoxifen treatment) association is shown in Figure 2A (p = 0.015). The lower expression of *TRAF1* is associated with fewer viable cells indicating higher endoxifen sensitivity. The same phenotype is also associated with hsa-let-7i expression at p = 0.006, with higher hsa-let-7i expression corresponding to higher cellular sensitivity to endoxifen (Figure 2B). Indeed, a significant negative correlation was observed between *TRAF1* and hsa-let-7i expressions (Figure 2C, p = 1×10^−4^). The CC genotype of rs4386686 is associated with lower expression of *TRAF1* (Figure 2D, p = 3×10^−5^). This same genotype is also associated with higher hsa-let-7i expression (Figure 2E, p = 0.02). As expected, the CC genotype is correlated with higher endoxifen sensitivity (p = 7×10^−5^, Figure 2F). Moreover, by taking advantage of the published drug response in 44 breast cancer cell lines [16], we found *TRAF1* expression is significant associated with –log_10_ (TAM GI_50_) in these breast cancer cell lines (Figure 2G).

In an effort to investigate whether identified genes and miRNAs are affecting TAM sensitivity only in the African derived cell lines, we cross-examined relationship between the expressions of 34 genes and 30 miRNAs identified in YRI and endoxifen sensitivity in 60 CEU (*Centre d'Etude du* Polymorphisme Humain, Utah) LCLs (reported previously in [10]). We found 8 genes and 4 miRNAs were also associated with endoxifen sensitivity in CEU, suggesting shared biological pathways in different ethnic groups. They are: *AMPD3, ITM2B, LARGE, RUFY4, SLC43A1, TNFAIP2, TRAF1, UAP1L1* and hsa-let-7i, hsa-miR-363, hsa-miR-519e*, hsa-miR-765. Closer examination in CEU to include only negative correlation between the endoxifen associated genes and miRNAs, we found a total of 4 associations with genes and miRNAs replicated, as shown in Table 2. Therefore, we performed functional validation for them in a breast cancer cell line.

**Table 2:** Examples of endoxifen sensitivity associated gene/miRNA expression identified in YRI and replicated in CEU LCLs.

SNP	miRNAs	genes	p-S1		p-S2		p-S3		p-S4		p-S5		p-S6	
			YRI	CEU	YRI	CEU	YRI	CEU	YRI	CEU	YRI	CEU	YRI	CEU
rs4386686	let-7i	*TRAF1*	1.5×10^−2^	3.3×10^−2^	6.5×10^−3^	7.3×10^−3^	6.3×10^−5^	NS	3.0×10^−5^	NS	2.0×10^−2^	NS	7.3×10^−5^	NS
rs4700416	let-7i	*TRAF1*	1.5×10^−2^	3.3×10^−2^	6.5×10^−3^	7.3×10^−3^	6.3×10^−5^	NS	3.0×10^−5^	NS	2.0×10^−2^	NS	7.3×10^−5^	NS
rs17104213	miR-363	*AMPD3*	4.0×10^−2^	2.7×10^−3^	2.0×10^−3^	2.6×10^−2^	1.9×10^−5^	3.9×10^−5^	3.0×10^−6^	NS	4.2×10^−2^	NS	6.4×10^−5^	NS
rs12915737	miR-363	*LARGE*	1.6×10^−2^	2.7×10^−2^	2.0×10^−3^	2.6×10^−2^	1.1×10^−5^	1.8×10^−5^	9×10^−5^	NS	3.1×10^−4^	NS	5.6×10^−5^	NS

p-S1, p-S2, p-S3, p-S4, p-S5 and p-S6 are short for p-values of each step, from step 1 to step 6. Step 1, the association between the phenotypes of endoxifen sensitivity and ∼13,000 gene/transcript cluster expressions; step 2, the association between the phenotypes of endoxifen sensitivity and 201 miRNA expressions; step 3, the negative correlation between mRNA and miRNA expressions from step 1 and step 2; step 4, the association between mRNA expressions from step 3 and SNP genotypes; step 5, the association between miRNA expression from step 3 and SNP genotypes from step 4; step 6, the association of SNP genotypes from step 5 and the phenotypes of endoxifen sensitivity. NS, not significant at p<0.05 threshold.

### Functional Validation of hsa-let-7i and *TRAF1* in Breast Cancer Cell Line ZR-75-1

**Figure 3:**
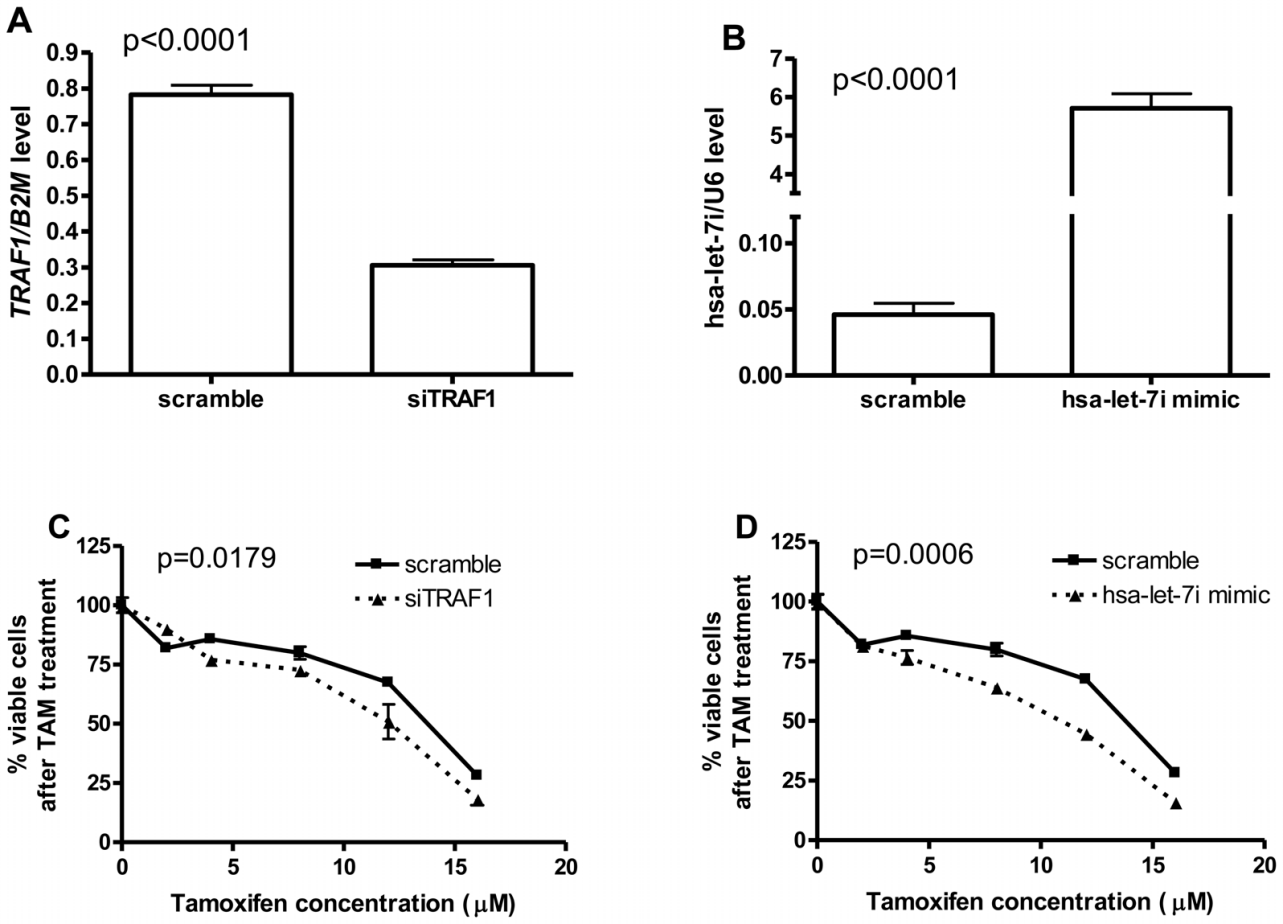
Increase of TAM sensitivity in breast cancer cell line ZR-75-1 with knock-down of *TRAF1* or overexpression of hsa-let-7i. **A**) Knock-down of *TRAF1* with siTRAF1 results in significantly decreased relative *TRAF1* expression characterized with qPCR; **B**) hsa-let-7i mimic transfection results in significantly increased relative let-7i expression characterized with qPCR; **C**) Increased TAM sensitivity in ZR-75-1 with knocking down of *TRAF1*; **D**) Increased TAM sensitivity in ZR-75-1 with over-expression of hsa-let-7i. P-values in panel A) and B) were calculated with paired t-test, while two-way ANOVA analysis was used to calculate p-values in panel C) and D). *B2M* and *RNU6* expressions were used as housekeeping to calculate the relative levels of *TRAF1* and hsa-let-7i expressions respectively.

To validate the roles of hsa-let-7i and *TRAF1* in TAM therapy, we knocked down *TRAF1* with siRNA, separately we also over-expressed hsa-let-7i with its mimic in a breast cancer cell line, ZR-75-1. The knock-down and overexpression experiments are successfully performed in ZR-75-1 (Figure 3A, 3B) with significantly decreased *TRAF1* and increased hsa-let-7i level, by comparing to the scramble controls after 24 hours of transfection (p<0.0001). Both inhibition of *TRAF1* and over-expression of hsa-let-7i result in significant increase of TAM sensitivity in ZR-75-1 cell line (Figure 3C, 3D, with the two-way ANOVA p-values 0.0179 and 0.0006, respectively).

## Discussion

In order to fill in the void of lack of study focusing in ethnic minority, we chose to quantitatively evaluate the cellular growth inhibition induced by endoxifen in HapMap YRI LCLs. An integrative omic model involved with genome-wide integrative analysis among 4 datasets (endoxifen cellular sensitivity, SNP genotype, mRNA and miRNA expressions) was employed to filter and identify SNPs, genes and miRNAs that are associated with each other but also correlated with endoxifen sensitivity. We identified 141 associations involved 50 SNPs, 34 genes and 30 miRNAs, all of which related to endoxifen sensitivity in these cell lines. Further functional validation confirmed the roles of hsa-let-7i and *TRAF1* affecting TAM sensitivity in a breast cancer cell line ZR-75-1, supported the values of our integrative omic analysis approach in the identification of novel biomarkers.

Ethnic differences were observed in breast cancer incidence, including ER-positive breast cancer, and treatment outcomes in Caucasian and African Americans [5]. Furthermore, studies focused on African breast cancer patients are lacking. We sought to identify pharmacogenomic biomarkers related to TAM therapy in African-derived samples. Interestingly, a cross-check of biomarkers identified in YRI with endoxifen sensitivity in CEU LCLs suggested some miRNAs and genes markers are shared in samples derived from both ethnic groups, such as hsa-let-7i, hsa-miR-363, *TRAF1*, and *AMPD3*; while others might be unique to African. hsa-let-7i has been reported to induce TAM sensitivity by down-regulating ER signaling pathway [17]. TRAF1, a TNF receptor associated factor, has been reported to interact with USP7 [18], a gene we have previously identified through a GWAS to affect TAM sensitivity in CEU samples [10]. This interaction was further supported in our investigation that *USP7* and *TRAF1* expressions is correlated in HapMap YRI LCLs (p = 0.0495). Our previous work showed that USP7, a ubiquitin specific protease, was related to TAM sensitivity in Caucasian derived LCLs and inhibition of *USP7* resulted in TAM resistance in 2 breast cancer cell lines [10]. We speculated this effect is achieved through cleaving ubiquitin from its target proteins, such as p53 and PTEN, and stabilize these proteins having the potential to promote cellular apoptosis. We hypothesize that USP7 can protect TRAF1 in the same way as it does to p53 and PTEN. Therefore, the stabilized TRAF1 might work as TNF receptor associated factor to inhibit apoptosis via NF-kB [19], [20]. The correlation of high *TRAF1* expression with high cellular resistance to endoxifen treatment observed in YRI LCLs, and the knocking down of *TRAF1* resulting in increased TAM sensitivity in breast cancer cell line both support this hypothesis.

Combined with our precious report, our GWAS discoveries of pharmacogenomic biomarkers with endoxifen treatment in both CEU and YRI LCLs seem to shed light on some important pharmacologic pathway involved in TAM sensitivity, such as the ubiquitin-mediated proteolysis. As a post-translational modification process, ubiquitination has been suggested to play an active role in apoptosis, DNA transcription, cellular differentiation, and others [21]–[23]. This process might control the abundance of many proteins related to cellular proliferation or apoptosis (such as p53, PTEN, and TRAF1) so that changes of their expressions might affect cellular sensitivity to TAM treatment.

In an effort to identify potentially ethnic group specific biomarkers, we compared the allele frequencies of the 50 SNPs initially identified in YRI to those in the CEU group. We found 7 SNPs (rs10015634, rs10753284, rs17104213, rs4073942, rs4386686, rs6811821 and rs7700034) showed more than 30% allele frequency differences. Not surprisingly, none of these SNP was found to be associated with TAM sensitivity in CEU samples, suggest that they might be unique genetic markers for TAM sensitivity in YRI samples.

Our study reported 30 miRNAs correlated with TAM sensitivity possibly through their negative effect on gene expression. Among them, we have functionally validated the role of hsa-let-7i in TAM sensitivity in a breast cancer cell line. Other miRNAs, such as hsa-miR-22, hsa-miR-18a, and hsa-miR-363, may also contribute to TAM sensitivity. Same may apply to the 34 identified genes. Although we did not choose to validate them, some of them have been suggested to be regulated by estrogen receptors, such as *EGR3*, *NAB2*, and *VEGFA*. As an early growth response protein, EGR3 functions as a transcription factor controlling biological rhythm. NAB2 is the direct target of EGR3 via protein interaction. NAB2 also works as transcription factor in nucleus to regulate cellular growth and differentiation [24], [25]. For *VEGFA*, an ER-binding element was located at ∼1.5 kb upstream of its transcription start site and found to be directly induced by estradiol [26]. These findings again suggested the utility of our integrative omic analysis in identifying genetic biomarkers with regard to drug sensitivity.

Since we measured growth inhibition as a way to quantify differentiated drug responses, all kinds of growth inhibitions, including endoxifen-induced and cellular intrinsic growth, would be reflected in the phenotypes. There is a possibility that some of the biomarkers reported in this work might not associate with the growth inhibition specifically induced by endoxifen. Further, we are fully aware that our model identifies inter-related SNPs, gene and miRNA expressions related to TAM sensitivity, SNP/miRNA/gene that associated with TAM sensitivity but not related to the other omic components are missed. However, by emphasizing the associations from multiple datasets, we expect to see much more reduced false discover rate in the final GWAS discoveries [27]. We anticipate that the biomarkers we reported in this paper should have better chance to be validated in additional cancer cell lines and in clinical trials.

By performing integrative omic analysis among drug sensitivity, SNP genotypes, gene expressions and miRNA expressions, we identified a set of SNPs, genes and miRNAs all associated with endoxifen-induced cellular sensitivity. Further testing of these biomarkers in a breast cancer cell line confirmed the roles of hsa-let-7i and *TRAF1* in TAM sensitivity. Overall, our search for genetic variants related to TAM sensitivity in YRI LCLs complemented our previous discovery in CEU LCLs. These findings suggested some TAM sensitivity biomarkers might be unique in African-derived samples while others are applicable in Caucasians as well.

## Supporting Information

Figure S1 **The overall strategy of using integrative omic approach to conduct association studies among drug sensitivity, SNP genotype, mRNA and miRNA expressions.** In step 1, genome-wide association study (GWAS) was performed between more than 13,000 gene/transcript cluster expression and 4 endoxifen phenotypes [log-transformed percent viable cell after 3, 5, 7, 10 μM endoxifen treatment] independently, with cutoff p<0.05. Step 2, GWAS was run between levels of 201 LCL-expressed miRNAs and 4 endoxifen phenotypes, with p<0.05 as the filtering criteria. Step 3, negative correlations between genes and miRNA identified in step 1 and 2 were examined by using the SCAN database. The threshold used was p≤10^−4^. In step 4, association between SNP genotype and gene expression passed step 3 filtering was examined by using SCAN database using cutoff of p≤10^−4^. Step 5, the associated SNPs identified from step 4 were further investigated for their association with miRNA expression identified from step 3 (p<0.05). Step 6, the SNPs associated with both gene (step 4) and miRNA (step 5) expressions were submitted for a GWAS analysis against each endoxifen sensitivity phenotype independently (p≤10^−4^).
(PPT)

Table S1 **141 unique associations of SNPs, miRNA and mRNA expressions that are significantly related to endoxifen sensitivity.**
(XLS)
