# Supplementary material for: Integrative “Omic” Analysis for Tamoxifen Sensitivity through Cell Based Models
Source: PLoS One. 2014 Apr 3;9(4):e93420. doi: 10.1371/journal.pone.0093420 (PMC3974759; doi:10.1371/journal.pone.0093420)
Supplement: Figure S1 — The overall strategy of using integrative omic approach to conduct association studies among drug sensitivity, SNP genotype, mRNA and miRNA expressions. In step 1, genome-wide association study (GWAS) was performed between more than 13,000 gene/transcript cluster expression and 4 endoxifen phenotypes [log-transformed percent viable cell after 3, 5, 7, 10 μM endoxifen treatment] independently, with cutoff p<0.05. Step 2, GWAS was run between levels of 201 LCL-expressed miRNAs and 4 endoxifen phenotypes, with p<0.05 as the filtering criteria. Step 3, negative correlations between genes and miRNA identified in step 1 and 2 were examined by using the SCAN database. The threshold used was p≤10−4. In step 4, association between SNP genotype and gene expression passed step 3 filtering was examined by using SCAN database using cutoff of p≤10−4. Step 5, the associated SNPs identified from step 4 were further investigated for their association with miRNA expression identified from step 3 (p<0.05). Step 6, the SNPs associated with both gene (step 4) and miRNA (step 5) expressions were submitted for a GWAS analysis against each endoxifen sensitivity phenotype independently (p≤10−4). (PPT) [file pone.0093420.s001.ppt]

## Slide 1
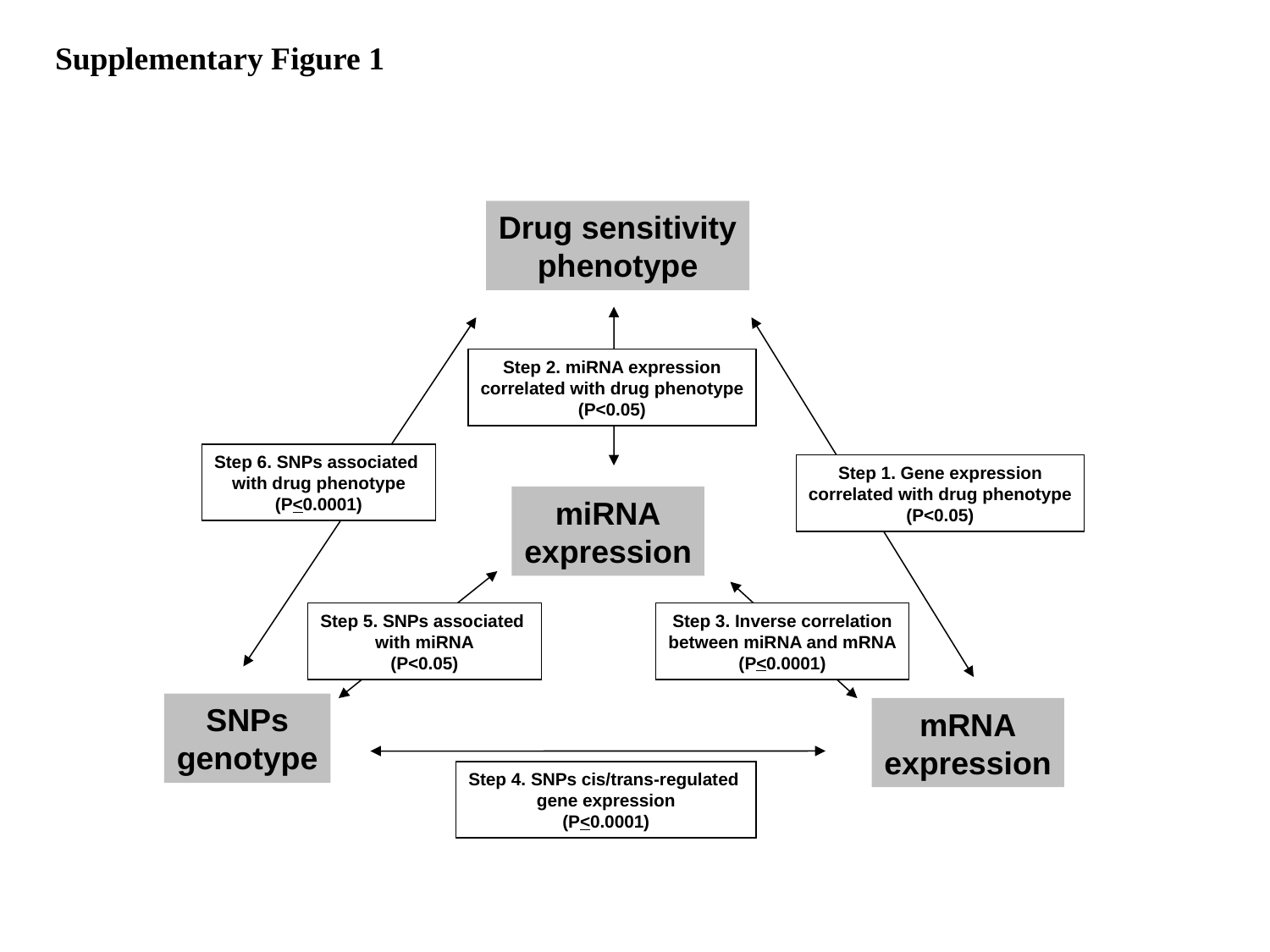

Supplementary Figure 1
Drug sensitivity
phenotype
Step 2. miRNA expression
correlated with drug phenotype
(P<0.05)
Step 6. SNPs associated
with drug phenotype
(P<0.0001)
Step 1. Gene expression
correlated with drug phenotype
(P<0.05)
miRNA
expression
Step 5. SNPs associated
with miRNA
(P<0.05)
Step 3. Inverse correlation
between miRNA and mRNA
(P<0.0001)
SNPs
genotype
mRNA
expression
Step 4. SNPs cis/trans-regulated
gene expression
(P<0.0001)
